# Resilience to changes in lake trophic state: Nutrient allocation into *Daphnia* resting eggs

**DOI:** 10.1002/ece3.5759

**Published:** 2019-10-29

**Authors:** Jana Isanta Navarro, Carmen Kowarik, Martin Wessels, Dietmar Straile, Dominik Martin‐Creuzburg

**Affiliations:** ^1^ Limnological Institute University of Konstanz Konstanz Germany; ^2^ Institute for Lake Research Langenargen Germany; ^3^ Present address: Eawag Swiss Federal Institute of Aquatic Science and Technology Dübendorf Switzerland

**Keywords:** anthropogenic impacts, Ephippia, Lake Constance, nutrient homeostasis, resilience

## Abstract

During past decades, many lakes underwent drastic human‐caused changes in trophic state with strong implications for population dynamics and food web processes. We investigated the influence of trophic state on nutrient allocation into *Daphnia* resting eggs. The production of resting eggs is an important survival strategy, allowing *Daphnia* to cope with unfavorable environmental conditions. Allocation of essential nutrients into resting eggs may crucially influence embryonic development and offspring survival and thus is of great ecological and evolutionary interest. The capacity of *Daphnia* to adjust the allocation of nutrients into resting eggs may depend on the dietary nutrient supply, which may vary with trophic state‐related changes in the phytoplankton community composition. Resting eggs were isolated from sediment cores taken from Lake Constance, a large prealpine lake with a distinct eutrophication and reoligotrophication history, and analyzed for elemental (carbon, nitrogen, and phosphorus) and biochemical (sterols and fatty acids) nutrients. Carbon allocation into *Daphnia* resting eggs continuously decreased over time, irrespective of changes in trophic state. The allocation of nitrogen into *Daphnia* resting eggs followed the changes in trophic state, that is, nitrogen concentrations in resting eggs increased with eutrophication and decreased again with reoligotrophication. The allocation of phosphorus, sterols and long‐chain polyunsaturated fatty acids, such as eicosapentaenoic acid, into *Daphnia* resting eggs did not change significantly over time. Changes in trophic state strikingly influenced all trophic levels in Lake Constance. However, nutrient allocation into *Daphnia* resting eggs was mostly resilient to changes in lake trophic state.

## Graphical abstract

The capacity of Daphnia to adjust the allocation of nutrients into resting eggs may depend on the dietary nutrient supply, which may vary with trophic state‐related changes in the phytoplankton community composition. Resting eggs were isolated from sediment cores taken from Lake Constance, a large prealpine lake with a distinct eutrophication and reoligotrophication history, and analysed for elemental (carbon, nitrogen, and phosphorus) and biochemical (sterols and fatty acids) nutrients. We show that the allocation of some nutrients into resting eggs is highly conserved (P, PUFA, and sterols) and mostly resilient towards changes in lake trophic state, while the allocation of other nutrients (C and N) seems to be less tightly regulated and thus affected by environmental changes.

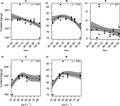

## 1 INTRODUCTION

Anthropogenic activities, such as industrialization, urbanization, and intensive agriculture, have resulted in increased nutrient loads and intense eutrophication of many lake ecosystem across the globe (Jeppesen et al., 2010; Smith, Tilman, & Nekola, 1999; Vitousek, Mooney, Lubchenco, & Melillo, 1997). In Lake Constance, a large prealpine lake with a distinct eutrophication and reoligotrophication history, total phosphorus concentrations during winter mixing (TP_mix_) increased more than tenfold from about 7 μg P/L in the 1950s to more than 80 μg P/L in the early 1980s (Güde, Rossknecht, & Wagner, 1998; IGKB, 2018). Subsequently, TP_mix_ decreased again to levels similar to the ones measured in the 1950s due to a substantial reduction in phosphorus loads (IGKB, 2018; Jochimsen, Kümmerlin, & Straile, 2013). Lake Constance offers unique possibilities to study trophic state‐related effects on ecosystem processes. Long‐term data are available for many abiotic and biotic parameters since the 1960s. In addition, annually laminated sediment cores and established depth‐related sediment age models (Wessels, Lenhard, Giovanoli, & Bollhöfer, 1995; Wessels, Mohaupt, Kümmerlin, & Lenhard, 1999) allow reconstructing the influence of changes in trophic state on the plankton community by analyzing plant and animal remains in sediment layers of different ages (Brede et al., 2009; Hairston et al., 1999).

The deposition of resting eggs by the freshwater keystone herbivore *Daphnia* is of particular interest. Resting eggs can be used to study temporal changes in the genetic architecture of *Daphnia* populations (Brede et al., 2009; Möst et al., 2015) and to apply resurrection ecology approaches (Decaestecker, Meester, & Mergeay, 2009; Frisch et al., 2014; Hairston et al., 1999). *Daphnia* can switch between asexual (parthenogenetic) and sexual reproduction. During parthenogenetic reproduction, females produce diploid eggs that develop directly into female offspring. However, the same female may produce diploid asexual eggs that develop into males or may produce up to two haploid resting eggs that require fertilization and then are enclosed in a protective shell (ephippium). Ephippia are shed with the old exoskeleton, dispersed with water currents, and finally sink to the bottom where they are partially deposited into the sediments. Resting eggs need to undergo a diapause before female offspring will hatch from them, which then start again reproducing parthenogenetically. In *Daphnia*, resting egg production is an important survival strategy, which is induced by high population densities and/or cues indicating deteriorating environmental conditions, such as declining food availability or photoperiod, or the presence of predator kairomones (Carvalho & Hughes, 1983; Kleiven, Larsson, Hobæk, & Hobaek, 1992; Ślusarczyk, 2001). Nutrient allocation into asexually produced subitaneous eggs has been shown already to significantly influence offspring growth and reproduction (Gliwicz & Guisande, 1992; Schlotz, Ebert, & Martin‐Creuzburg, 2013; Sperfeld & Wacker, 2015). Nutrient allocation into sexually produced resting eggs has been also studied (Abrusán, Fink, & Lampert, 2007; Putman, Martin‐Creuzburg, Panis, & Meester, 2015) but potential effects on offspring performance have not been demonstrated yet. It can be assumed, however, that nutrient allocation into resting eggs is also optimized in order to ensure embryonic development and offspring survival. Changes in lake trophic state are associated with changes in the availability of elemental nutrients required for growth and reproduction of zooplankton (Hartwich, Martin‐Creuzburg, Rothhaupt, & Wacker, 2012; Sterner & Elser, 2002). The C:P and C:N ratios of lake seston is usually higher at oligotrophic than eutrophic conditions (Elser, Frost, Kyle, Urabe, & Andersen, 2002; Hessen, 2006). Growth rate in zooplankton is tightly linked to elemental nutrient ratios that are homeostatically regulated (Sterner & Elser, 2002).

Changes in lake trophic state can also result in fundamental shifts in the phytoplankton community composition (Jochimsen et al., 2013), which may also affect the performance of zooplankton through changes in the availability of essential biochemicals, such as long‐chain polyunsaturated fatty acids (PUFA) and sterols (Hartwich et al., 2012; Martin‐Creuzburg & Merkel, 2016; Müller‐Navarra et al., 2004). Under oligotrophic conditions, the phytoplankton community is often dominated by cryptophytes and diatoms, while green algae and cyanobacteria typically gain in importance under eutrophic conditions (Sommer et al., 2012; Sommer, Gliwicz, Lampert, & Duncan, 1986). Cryptophytes and diatoms are rich in long‐chain PUFA and thus are commonly considered as high‐quality food for zooplankton (Ahlgren, Lundstedt, Brett, & Forsberg, 1990; Carotenuto, Wichard, Pohnert, & Lampert, 2005). In comparison, green algae are of intermediary food quality due to a deficiency in C20‐PUFA (von Elert, 2002). Cyanobacteria are of poor food quality for zooplankton because of morphological properties, the production of harmful secondary metabolites, and the lack of sterols and long‐chain PUFA (de Bernardi & Giussani, 1990; von Elert, Martin‐Creuzburg, & Coz, 2003; Martin‐Creuzburg, Elert, & Hoffmann, 2008; Wilson, Sarnelle, & Tillmanns, 2006).

Dietary deficiencies in sterols and long‐chain PUFA are well‐known to severely constrain growth and reproduction of *Daphnia* (von Elert, 2002; Hartwich et al., 2012; Martin‐Creuzburg et al., 2008). Both lipid classes are indispensable as structural components of cell membranes and as precursors for other bioactive molecules, such as ecdysteroids (sterols), which in arthropods are involved in the process of molting, and eicosanoids (PUFA), which play crucial roles as signaling molecules in reproduction, immunity, and other physiological processes (Grieneisen, 1994; Stanley, 2000). Sterol auxotrophy is widespread among invertebrates, and it is generally accepted that all arthropods lack the capacity to synthesize sterols de novo and thus need to convert the sterols provided in their diet into cholesterol, the main body sterol of most animals (Goad & Withers, 1982; Martin‐Creuzburg & von Elert, 2009; Martin‐Creuzburg, Oexle, & Wacker, 2014). Cyanobacteria do not contain sterols, and it has been shown that daphnids feeding on a diet containing high percentages of cyanobacteria exhibit a reduced sterol content and reduced fitness (Martin‐Creuzburg & von Elert, 2004; Martin‐Creuzburg, Wacker, & Elert, 2005). Therefore, the predominance of cyanobacteria in lake seston may lead to a sterol limitation in *Daphnia*, which may affect the allocation of sterols into their resting eggs. In addition to sterols, most arthropods require a dietary source of PUFA. The two C18‐PUFA linoleic acid (18:2n‐6; LIN) and α‐linolenic acid (18:3n‐3; ALA) are commonly considered to be essential for most animals (Arts, Brett, & Kainz, 2009). Once taken up with the food, they may serve as precursors for long‐chain PUFA, such as arachidonic acid (20:4n‐6; ARA) and eicosapentaenoic acid (20:5n‐3; EPA), but the rates of elongation and desaturation are usually very low (Weers & Gulati, 1997; Weers, Siewertsen, & Gulati, 1997), as indicated, for instance, by the beneficial effects of ARA and EPA supplementation on growth and reproduction of *Daphnia* (von Elert, 2002; Martin‐Creuzburg, Wacker, & Basen, 2010).

The diet of the nonselective filter‐feeder *Daphnia* consists of phytoplankton, bacteria, protozoa, and detritus in varying proportions. *Daphnia* have limited capacities to modify dietary PUFA and their body fatty acid composition thus typically reflects that of their food source (Brett & Müller‐Navarra, 1997; Brett, Müller‐Navarra, Ballantyne, Ravet, & Goldman, 2006; Ravet, Brett, & Müller‐Navarra, 2003). Furthermore, female *Daphnia* allocate substantial amounts of fatty acids into their subitaneous eggs in ratios similar to what is found in the food (Schlotz et al., 2013; Wacker & Martin‐Creuzburg, 2007). The fatty acid composition of sexually produced resting eggs also reflects that of the maternal food, albeit with small but potentially important modification. Compared with asexual eggs, sexual eggs seem to contain higher amounts of PUFA, especially EPA, even when produced on a diet deficient in long‐chain PUFA (Abrusán et al., 2007; Putman et al., 2015). Wacker and Martin‐Creuzburg (2007) have shown that cholesterol allocation into asexual eggs is more homeostatic than EPA allocation at low food quality. Allocation of sterols into *Daphnia* resting eggs has not been studied yet. Overall, nutrient allocation into *Daphnia* resting eggs is poorly understood. Daphnids may either unselectively transfer dietary nutrients into their resting eggs or may selectively allocate single, high‐quality nutrients into their resting eggs to ensure embryonic development and to increase the chance of offspring survival. In any case, nutrient allocation is most likely affected by food quantity and quality. To assess the impact of trophic state on nutrient allocation, resting eggs of *Daphnia* were isolated from the sediment egg bank of Lake Constance and analyzed for elemental (carbon, nitrogen, and phosphorus) and biochemical (sterols and fatty acids) nutrient concentrations. We hypothesized that nutrient allocation into *Daphnia* resting eggs is affected by temporal changes in trophic state and the associated changes in food quantity (i.e., phytoplankton biovolume) and quality (i.e., relative proportions of phytoplankton taxonomic groups).

## 2 METHODS

### 2.1 Study site

The study was conducted in Lake Constance, a large prealpine lake situated in southern Germany at the borders to Switzerland and Austria. With a maximum depth of 251 m and a surface area of 473 km^2^ (Upper Lake Constance), Lake Constance is one of the largest and deepest lakes in central Europe. Phytoplankton was sampled twice a month at the deepest part of the lake within the Lake Constance long‐term monitoring program of the IGKB (International Commission for the Protection of Lake Constance). Cell concentrations were converted to biovolumes using species‐specific cell volumes. More details about sampling and counting procedures can be found in Jochimsen et al. (2013). For this study, the biovolumes of cyanobacteria, chlorophytes, diatoms, and cryptophytes as a proportion of total phytoplankton biovolume were calculated for each sampling date and averaged across the months May to October of each study year (1965–2007).

**Figure 1:**
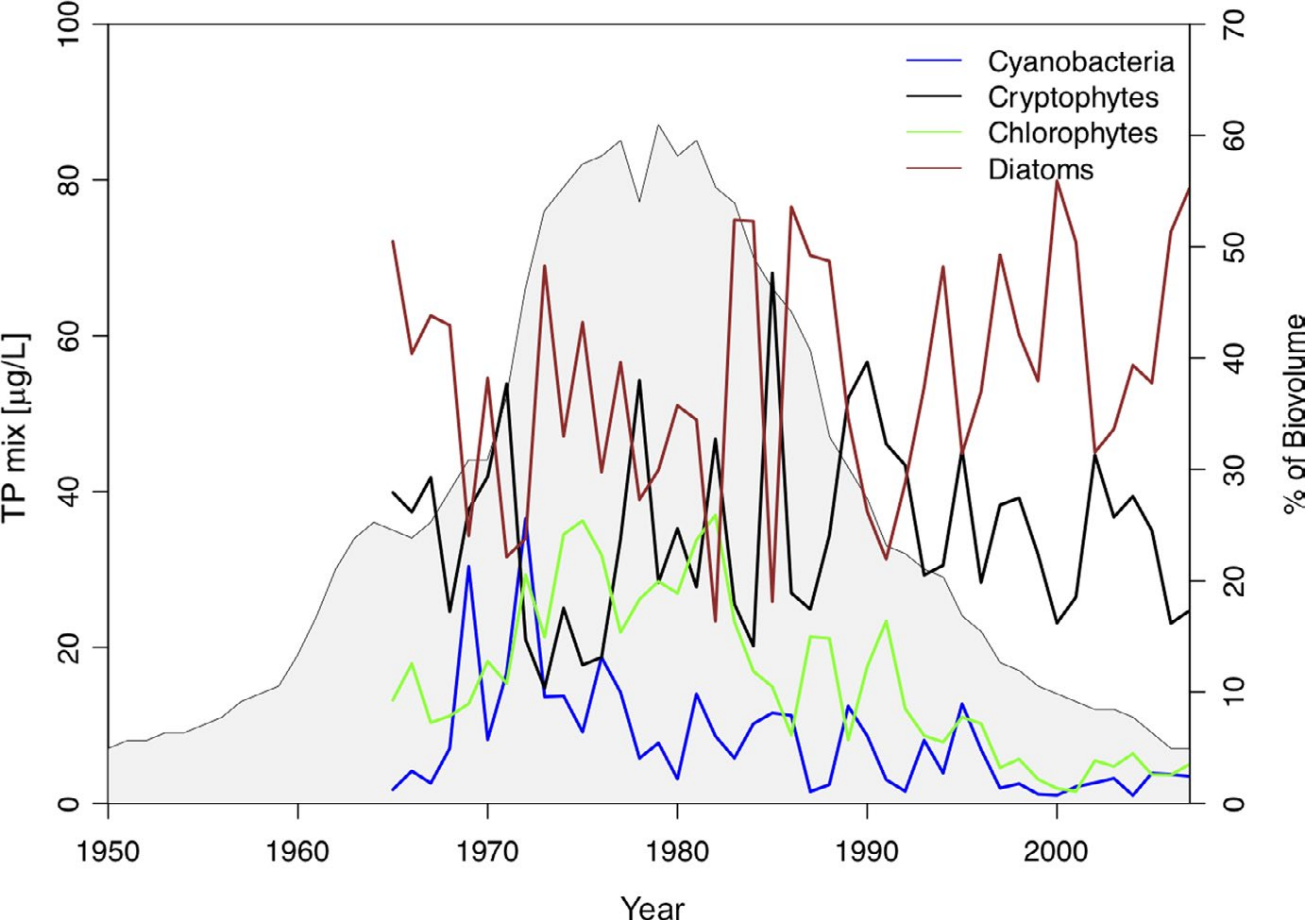
Temporal changes in total phosphorus concentration (TP_mix_) in Lake Constance, reaching highest concentrations during peak eutrophication in the late 1970s, and temporal changes in the relative contribution of the four main phytoplankton groups (% of total biovolume during summer month May–October) over past decades. Diatoms (brown) and cryptophytes (black) dominated the phytoplankton community over the entire time span. The highest cyanobacterial biomass was recorded during peak eutrophication

Lake Constance underwent a distinct eutrophication and reoligotrophication history and these changes in trophic state also led to shifts in the phytoplankton community compositions (Jochimsen et al., 2013; Wessels et al., 1999). During summer months (May–October), the share of chlorophytes and cyanobacteria increased with eutrophication and represented about 25%–40% of total phytoplankton biomass at peak eutrophication (1970–1985; Figure 1). However, even during peak eutrophication, diatoms remained the dominant algal group.

Eutrophication of Lake Constance resulted in changes in the *Daphnia* species composition (Brede et al., 2009; Straile & Geller, 1998). In the first half of the last century, only *Daphnia longispina* (formerly termed *Daphnia hyalina*) occurred in Upper Lake Constance. In the course of eutrophication, *Daphnia galeata* invaded Upper Lake Constance in the late 1950s (Straile & Geller, 1998). Nowadays, both species and their hybrids are found in the lake, together with a third species (*Daphnia cucullata*) that was detected in Upper Lake Constance in 2014 and became dominant in recent years.

### 2.2 Sediment cores

Sediment cores were taken in spring and autumn 2017 in the Bay of Friedrichshafen in Upper Lake Constance (47°36′26.51″N 9°27′45.82″E). Using a multicorer, a total of 21 sediment cores were taken (three sediment cores at a time) in a depth of approximately 185 m. The cores were split into halves and stored at 4°C in the dark. Sediment cores were partitioned into 5‐year spanning sections, ranging from 2010 to 1945. The age of the sediment layers was determined following Wessels et al., (1995), Wessels et al., (1999).

### 2.3 Isolation of resting eggs

The sediment samples were sieved through a 250 µm mesh, and ephippia were isolated under a stereomicroscope (Zeiss Stemi 2000‐C). Ephippia were opened and the resting eggs were isolated, washed twice with ultrapure water and stored at −80°C until further analysis. Only unimpaired eggs were used for the analyses: 80–100 eggs for one sterol, fatty acid and C/N analysis, respectively, and 20 eggs for one phosphorus analysis. Isolated eggs were randomly distributed between the respective analyses. The number of sediment cores required to obtain sufficient resting eggs from one particular time span varied between 3 and 21, because the deposition of ephippia was much higher during the eutrophic phase than during the oligotrophic phases. Sediment older than 1945 did not provide sufficient numbers of ephippia containing unimpaired resting eggs for subsequent analysis.

### 2.4 Elemental nutrient analysis

Resting eggs deposited between 1950 and 2005 were analyzed for carbon (C) and nitrogen (N) using a CN analyzer (EuroEA3000; HEKAtech GmbH). Resting eggs were collected in pre‐weighted tin shells and dried for 24 hr at 50°C prior to analysis. For analysis of phosphorus (P), resting eggs were digested using a solution of 10% potassium peroxydisulfate and 1.5% sodium hydroxide for 1 hr at 121°C and the soluble reactive phosphorus was then determined using the molybdate‐ascorbic acid method (Greenberg, Trussell, Clesceri, & Franson, 1985). The data are presented relative to egg dry weight (ng/µg DM; means ± 1 *SE*) and as molar ratios.

### 2.5 Fatty acid and sterol analysis

For the analysis of fatty acids and sterols, resting eggs were deposited in pre‐weighted aluminium boats, stored at −80°C, freeze‐dried, weighted again for dry weight determination, transferred into 7 ml of a mixture of dichloromethane: methanol (2:1, v:v), and stored at −20°C. Prior to fatty acid and sterol analyses, internal standards were added (C23:0 ME into fatty acid samples and 5α‐cholestane into sterol samples). After 5 min in an ultrasound bath and subsequent centrifugation (3,500 *g*, 5 min), total lipids were extracted three times from resting eggs with dichloromethane: methanol (2:1, v:v). The pooled particle‐free extracts were evaporated to dryness using nitrogen and transesterified with 3 mol/L methanolic HCL (60°C, 15 min) for the analysis of fatty acids or saponified with 0.2 mol/L methanolic KOH (70°C, 1 hr) for the analysis of sterols. Afterward, fatty acid methyl esters (FAMEs) were extracted three times with 2 ml of iso‐hexane and neutral lipids were separated into iso‐hexane: diethyl ether (9:1, v:v). The lipid‐containing fractions were pooled, evaporated to dryness under nitrogen, and resuspended in 10 µl iso‐hexane. Lipids were analyzed and quantified by gas chromatography on a HP 6890 gas chromatograph (GC) equipped with a flame ionization detector (FID) and a DB‐225 (30 m × 0.25 mm i.d. × 0.25 µm film; J&W Scientific) capillary column to analyze FAMEs, or with a HP‐5 (30 m × 0.25 mm i.d. × 0.25 µm film; Agilent) capillary column to analyze sterols. Lipids were identified using a gas chromatograph‐mass spectrometer (GC‐MS; 5975C inert MSD; Agilent Technologies). The C‐24 stereochemistry of epi‐/brassicasterol and sito‐/clionasterol, which were found in trace amounts in the resting eggs, could not be identified with certainty and thus is not specified here. Details of GC configurations for the analysis of fatty acids are given in Martin‐Creuzburg et al. (2010) and for the analysis of sterols in Martin‐Creuzburg and Merkel (2016). The lipid data are presented relative to egg dry weight (ng/µg DM; means ± 1 *SE*).

### 2.6 Data analysis

A generalized additive model (GAM) (Wood, 2006) was applied to analyze changes in nutrient allocation into resting eggs in relation to time, total phosphorus (TP_mix_), and phytoplankton taxonomic group composition using the “mgcv” (Wood, 2018) and “ggplot” (Wickham, 2016) packages and the PCA was conducted using the “ggfortify” (Tang, Horikoshi, & Li, 2016) package implemented in R (R Core Team, 2016). GAMs were used because they allow for potential nonlinear dynamics and relationships to independent variables. However, to avoid the establishment of overly complex GAMs, we set the dimension of the basis of the smooth to *k* = 5. TP_mix_ and late spring/summer phytoplankton data (May–October) were extracted from the long‐term data set available for Lake Constance; they were averaged over the 5‐year spanning time periods, and the resulting means were used for GAM. Phytoplankton late spring/summer means were used since *Daphnia* resting eggs normally are produced within this time period in Lake Constance (Jankowski & Straile, 2004). Analyses were performed for each trait (egg dry weight, elements, fatty acids, and sterols) separately; they were correlated with time, total phosphorus concentration, and summer phytoplankton biovolume as well as main phytoplankton taxonomic groups. However, if no significant changes over time were detected (*p* > .05), further correlations with total phosphorus, summer phytoplankton biovolume, and main phytoplankton taxonomic groups were not considered and are not shown here. For a summary of structure and results of GAMs please see Table S1.

## 3 RESULTS

### 3.1 Egg dry weight

**Figure 2:**
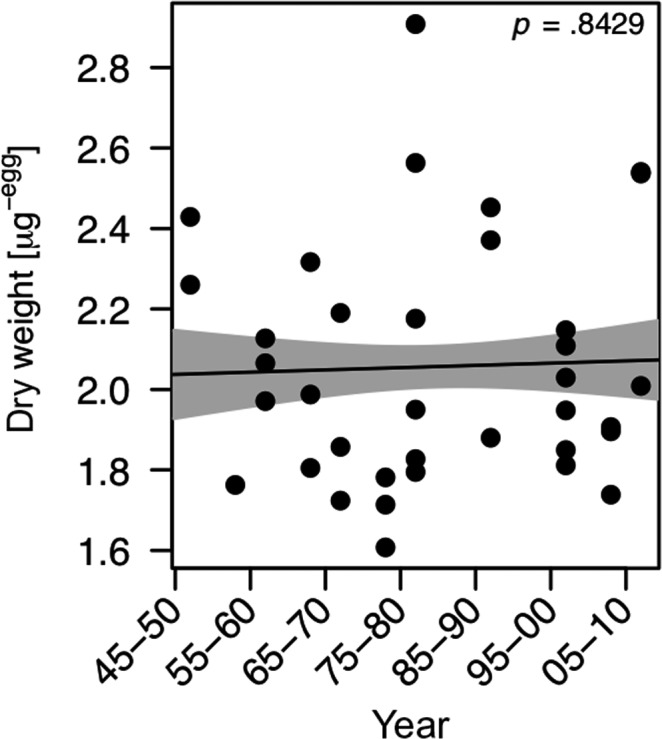
Temporal changes in mean dry weight of resting eggs isolated from sediment layers of different ages (1945–2010). The same eggs were then used for subsequent nutrient analyses. Each replicate consisted of 80–100 eggs. Data from 1945 to 1955 represent only two replicates due to scarcity of resting eggs in these sediment layers. The solid line represents the fit for the generalized additive model (GAM). The gray areas represent the ±1 SE of the fit

The dry weight of resting eggs from twelve 5‐year time spans between 1945 and 2010 fluctuated around 2 µg per egg and did not reveal significant changes over time (Figure 2) (GAM, *p* > .05).

### 3.2 Elements

**Figure 3:**
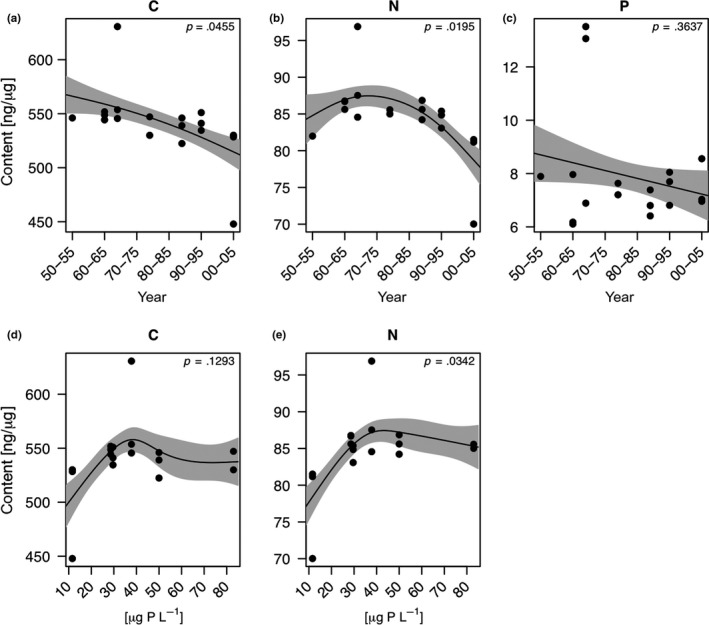
Temporal changes in the allocation of elemental nutrients (C, N, and P) into *Daphnia* resting eggs (a–c). Panels d and e depict the relationship between elemental nutrients (C and N) and total phosphorus concentrations (TP_mix_) in Lake Constance. Relationships between nutrient concentrations in resting eggs and TP_mix_, phytoplankton biovolume, and phytoplankton taxonomic are only depicted when the model revealed significant changes in nutrient concentrations over time. The solid line represents the fit for the generalized additive model (GAM). The gray areas represent the ±1 *SE* of the fit

**Figure 4:**
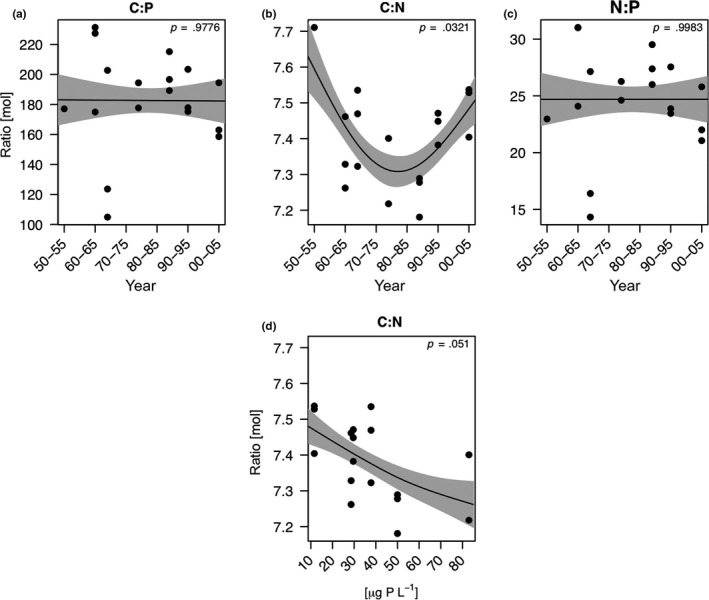
Temporal changes in the allocation of elemental nutrient ratios (C:P, C:N, and N:P) into *Daphnia* resting eggs (a–c). Panel d depicts the relationship between elemental nutrients (C and N) and their ratio and phosphorus concentrations (TP_mix_) in Lake Constance. Relationships between nutrient concentrations in resting eggs and TP_mix_, phytoplankton biovolume, and phytoplankton taxonomic are only depicted when the model revealed significant changes in nutrient concentrations over time. The solid line represents the fit for the generalized additive model (GAM). The gray areas represent the ±1 *SE* of the fit

The allocation of carbon into resting eggs significantly decreased with time (GAM, *p* < .05; Figure 3a). Nitrogen allocation was highest during eutrophic times and then decreased concurrently with TP_mix_ (Figure 3b). Phosphorus allocation into resting eggs did not change significantly over time and did not correlate with TP_mix_ (Figure 3c). The C:P and N:P ratios in the eggs also did not change significantly over time (Figure 4a,c). For C:N ratios, our model suggests a significant change both with time (Figure 4b) and in relation to TP_mix_ (Figure 4d). The C:N ratio in resting eggs was lowest during peak eutrophication (1970–1985), that is, at highest phosphorus concentrations in Lake Constance (Figure 4d).

### 3.3 Sterols

**Figure 5:**
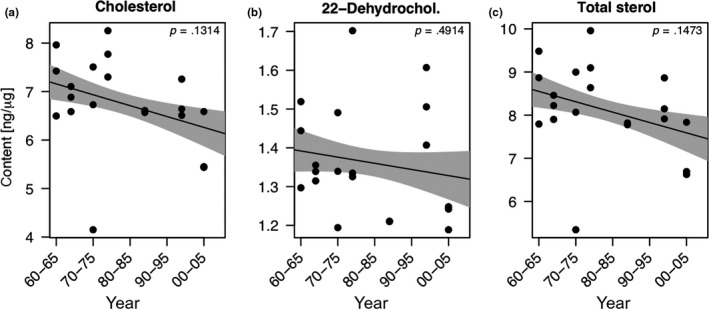
Temporal changes in the allocation of sterols (cholesterol, 22‐dehydrocholesterol, and total sterol content) into *Daphnia* resting eggs (a–c). Total sterol content is the sum of cholesterol and 22‐dehydrocholesterol. The solid line represents the fit for the generalized additive model (GAM). The gray areas represent the ±1 *SE* of the fit

Cholesterol was the predominant sterol found in resting eggs, it was accompanied by lower amounts of 22‐dehydrocholesterol and trace amounts of a few other sterols (campesterol, epi‐/brassicasterol, and sito‐/clionasterol) which were not further evaluated. The total sterol content (8.13 ± 1.05 ng/µg), the cholesterol content (6.76 ± 0.94 ng/µg), and the 22‐dehydrocholesterol content (1.36 ± 0.14 ng/µg) did not change over time. The GAM model revealed that the allocation of sterols into *Daphnia* resting eggs was unrelated to the temporal changes in phosphorus concentrations (GAM, *p* > .05; Figure 5), to summer phytoplankton biovolume, and the relative abundance of taxonomic phytoplankton groups (data not shown).

### 3.4 Fatty acids

**Figure 6:**
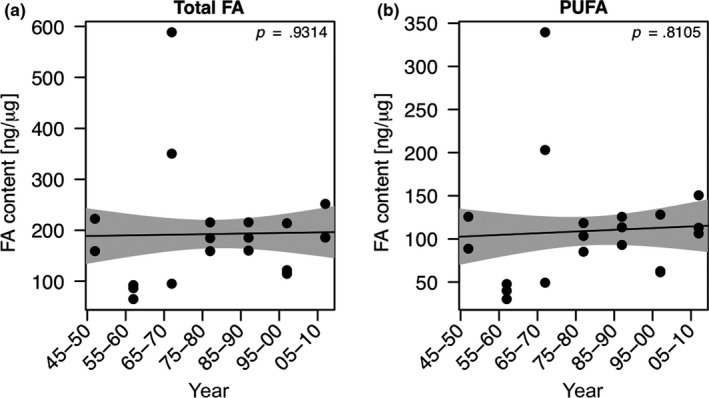
Temporal changes in the allocation of fatty acids (total fatty acids and polyunsaturated fatty acids) into *Daphnia* resting eggs (a and b). The solid line represents the fit for the generalized additive model (GAM). The gray areas represent the ±1 *SE* of the fit

**Figure 7:**
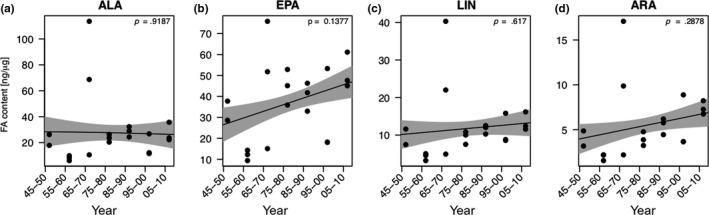
Temporal changes in the allocation of polyunsaturated fatty acids (α‐linolenic acid, eicosapentaenoic acid, linolenic acid, and arachidonic acid) into *Daphnia* resting eggs (a–d). The solid line represents the fit for the generalized additive model (GAM). The gray areas represent the ±1 *SE* of the fit

Allocation of total fatty acids into *Daphnia* resting eggs did not change significantly over the past decades and the GAM model did not reveal significant relationship (Figure 6a). Correlations of allocation of total fatty acids into *Daphnia* resting eggs was unrelated to the temporal changes in phosphorus concentrations, to summer phytoplankton, and the relative abundance of taxonomic phytoplankton groups (data not shown). Taking only PUFA into consideration, our model suggests that there was no significant change in total PUFA allocation over time (Figure 6b). The concentrations of α‐linolenic acid (ALA; C18:3n‐3), eicosapentaenoic acid (EPA; C20:5n‐3), linoleic acid (LIN; C18:2n‐6), and arachidonic acid (ARA; C20:4n‐6) did not change significantly over time (Figure 7a–d). Likewise, the fatty acid dynamics did not show any clear relationships with temporal changes in the concentrations of different algal groups (i.e., diatoms, cryptophytes, green algae, and cyanobacteria) extracted from the long‐term data set (data not shown).

**Figure 8:**
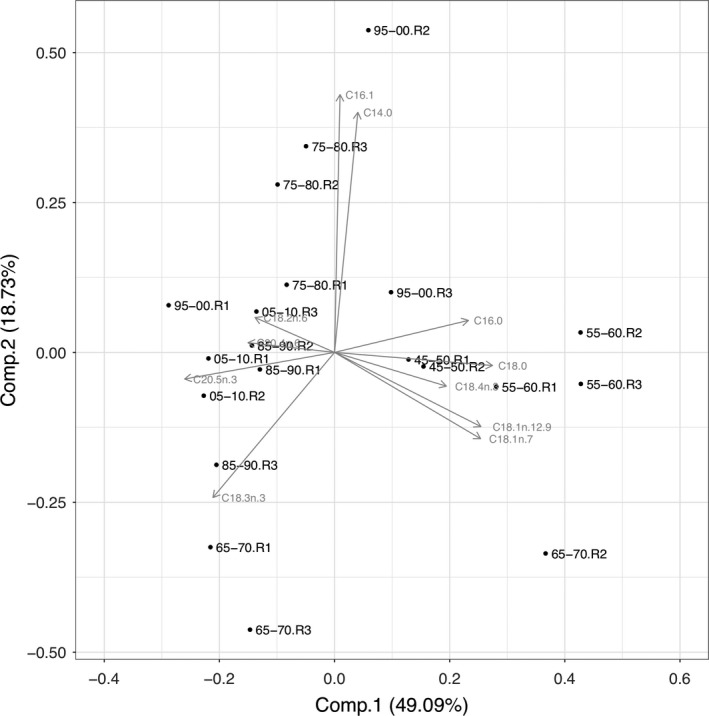
Principal component analysis (PCA) of the fatty acid profiles of resting eggs from 1945 to 2010. The PCA was calculated using all fatty acids that made up more than 5% of total fatty acids content. PC1 explained 49.09% and PC2 18.73% of total variation. Replicates per time zone are indicated with R1‐3

Principal component analysis (PCA) of fatty acid profiles (all fatty acids >5% of total fatty acids included) projected most of the variance of the multivariate data set on the first two principal components; PC1 explained 49.09% and PC2 explained 18.73% of variation. The PCA did, however, not reveal a clear general pattern regarding PUFA allocation. The resting egg samples from 1945 to 1960 (but also one sample from 1965 to 1970) were characterized by higher contributions of C16 and some C18 FAs. (Figure 8), whereas the years 2005–2010 showed lower contributions of those FAs. Likewise, the PCA did not clearly separate resting egg samples from the period 1975 to 2010, mainly because samples from 1995 to 2000 showed a high variability in PUFA composition (Figure 8). Resting eggs scores on PC1 differed significantly between resting eggs produced from 1945 to 1960 and resting eggs from all other time spans (ANOVA on Ranks, *p* = .003).

## 4 DISCUSSION

The human‐caused increase in phosphorus (P) and nitrogen (N) concentrations in naturally oligotrophic Lake Constance resulted in an increase in total phytoplankton biomass and changes in phytoplankton community composition (Güde & Straile, 2016; Jochimsen et al., 2013). Despite the striking changes in TP_mix_, P concentrations in *Daphnia* resting eggs did not change with time and did not reveal a significant relationship with TP_mix_. According to common practice, trophic state of Lake Constance was assessed using total P concentrations measured during winter circulation (TP_mix_), while resting eggs were most likely produced during the growing season (Jankowski & Straile, 2003, 2004). Dissolved P concentrations can be strongly depleted in the epilimnion during summer stratification, resulting in high seston C:P ratios. Thus, resting eggs may have been produced during low P concentrations even during peak eutrophication, which partially may explain why P concentrations in *Daphnia* resting eggs did not follow the temporal changes in TP_mix_. It should be noted, however, that total phosphorus concentrations during winter circulation correlate well with summer phosphorus concentrations in Lake Constance (Güde & Straile, 2016), implying that resting eggs were produced at varying dietary P concentrations. The resistance of a consumer's elemental body composition to changes in the elemental composition of its food is commonly referred to as homeostasis (Sterner & Elser, 2002); and *Daphnia* have been shown to regulate their P body content homeostatically within certain limits (DeMott, Pape, & Tessier, 2004; DeMott & Tessier, 2002; Sterner & Elser, 2002). Considering that the *Daphnia* resting eggs were most likely produced at varying seston C:P ratios during summer, our finding suggests that P allocation into *Daphnia* resting eggs is also regulated homeostatically. In past 65 years, *Daphnia* from Lake Constance may thus have been able to maintain P homeostasis in their resting eggs despite substantial changes in P availability.

Our model suggests that carbon (C) allocation into *Daphnia* resting eggs significantly decreased over time, which cannot be explained with temporal changes in TP_mix_ or phytoplankton biomass (i.e., food quantity). The C data set was characterized by one very high data point in the late 1960s and one very low data point in the early 2000s, raising the question of whether this correlation is meaningful. However, the model was still significant when these two data points were removed, suggesting that carbon allocation into *Daphnia* resting eggs actually decreased over time. The putative decrease in carbon allocation over time could not be directly linked to changes in trophic state (i.e., TP_mix_, food quantity, and food quality).

In contrast to P, total N concentrations in Lake Constance increased constantly over the past decades (Güde & Straile, 2016). Nonetheless, allocation of N into *Daphnia* resting eggs increased with eutrophication and decreased again after peak eutrophication (1970–1985) to values similar to the ones measured before eutrophication. Thus, N allocation into *Daphnia* resting eggs seems to be independent of temporal changes in N concentrations in the lake and rather related to other unknown influences linked to eutrophication. The temporal changes in N concentrations in *Daphnia* resting eggs were reflected in temporal changes in C:N ratios, while C:P and N:P ratios did not change significantly over time. Whether *Daphnia* are actually capable of regulating elemental nutrient ratios in their resting eggs homeostatically remains to be tested experimentally. Overall, however, our data suggest that the allocation of elemental nutrients into *Daphnia* resting eggs is mostly resilient to changes in trophic state.

Resting eggs from all time periods contained cholesterol as the principal sterol, which was accompanied by lower amounts of 22‐dehydrocholeserol. Cholesterol is the main body sterol of most animals, including *Daphnia* (Martin‐Creuzburg et al., 2014). Cholesterol is also the predominant sterol in asexually produced subitaneous eggs of *Daphnia* (Wacker & Martin‐Creuzburg, 2007), and it is thus not surprising to find cholesterol also in *Daphnia* resting eggs. 22‐dehydrocholesterol might be an intermediate in the conversion of dietary phytosterols, like brassicasterol or stigmasterol, into cholesterol (Martin‐Creuzburg et al., 2014). The fact that 22‐dehydrocholesterol was detected in almost all resting egg samples implies that this sterol has specific physiological functions during embryonic development, a topic requiring further investigation. Sterol allocation into resting eggs was resilient toward changes in lake trophic state and the concurrent change in phosphorus concentration. We expected to find reduced sterol contents in resting eggs deposited during eutrophic conditions, because of a higher share of cyanobacteria in the phytoplankton. Cyanobacteria do not contain sterols and a higher share of cyanobacteria thus reduces the availability of sterols for zooplankton grazers. Based on annual averages, the contribution of cyanobacteria to total phytoplankton biomass never exceeded 13.5% (1972), and regarding summer means (May–October), cyanobacteria never represented more than 25.6% (1972) of total phytoplankton biomass, though their relative contribution was presumably much higher during the peak of cyanobacterial blooms (Güde & Straile, 2016). Supplementation experiments with nontoxic cyanobacteria suggest that *Daphnia* are limited by sterols when at least 30% of the total provided carbon is represented by cyanobacteria (von Elert et al., 2003; Martin‐Creuzburg et al., 2005; Schlotz, Pester, Freese, & Martin‐Creuzburg, 2014). It is thus possible that phytoplankton in Lake Constance always provided enough phytosterols that could be metabolized into cholesterol to prevent a limitation by sterols and to ensure sufficient cholesterol allocation into the resting eggs. Sterol allocation into asexually produced subitaneous eggs seems to be regulated more or less homeostatically in *Daphnia* (Wacker & Martin‐Creuzburg, 2007), and the data we provide here suggest that this also the case for sexually produced resting eggs. Besides cholesterol and 22‐dehydrocholeserol, epi‐/brassicasterol, sito‐/clionasterol, and campesterol were found in trace amounts in the resting eggs. Epi‐/brassicasterol is typically found in cryptophytes and some diatoms (Martin‐Creuzburg & Merkel, 2016). The presence of epi‐/brassicasterol in resting eggs of all time periods thus implies that the maternal diet always included cryptophytes and/or diatoms, which are of superior food quality for freshwater zooplankton in terms of sterols and PUFA (Martin‐Creuzburg & von Elert, 2009). Sito‐/clionasterol can be found in some chlorophytes, for example, in some *Chlorella* species, and campesterol has been found in many freshwater algae (Martin‐Creuzburg & Merkel, 2016; Nishimura & Koyama, 1977). These sterols are presumably not specific for a particular group of algae and thus unsuitable as trophic markers. Hence, the occurrence of these sterols in resting eggs does not provide additional information on the potential composition of the maternal diet on which the resting eggs were produced.

The PCA of fatty acid profiles did not clearly separate resting eggs produced during oligotrophic and eutrophic conditions, but the resting eggs deposited between 1945 and 1960 roughly grouped together, suggesting that the allocation of fatty acids into resting eggs changed over time either because of changes in the bioavailability of fatty acids or interspecific differences in fatty acid allocation due to the invasion of *D. galeata* (see below). The separation of resting eggs from 1945 to 1960 was driven by C18 fatty acids. Regarding the allocation of physiologically important PUFA, we did not find significant relationships with time, TP_mix_, total phytoplankton biovolume, or the proportions of the different taxonomic phytoplankton groups (data not shown). In *Daphnia*, body fatty acid profiles mostly reflect the fatty acid profiles of their food source, indicating that the capacity to modify dietary fatty acids and to maintain certain PUFA concentration in the body is limited (Brett et al., 2006). Our finding that PUFA concentrations in *Daphnia* resting eggs did not change over time suggests some sort of homeostatic regulation, especially in the light of temporal changes in phytoplankton community composition and thus dietary PUFA availability. Since chlorophytes and cyanobacteria are deficient in long‐chain PUFA and typically benefit from eutrophication, we expected to find less long‐chain PUFA in resting eggs deposited during eutrophic times. However, even though the biomass of cyanobacteria and chlorophytes increased with eutrophication, the share of both phytoplankton groups together never exceeded 50% of total summer phytoplankton biomass, which may explain why we did not find significant changes in PUFA allocation over time. Thus, we conclude that the seston of Lake Constance always provided high proportions of phytoplankton taxa rich in long‐chain PUFA, even at peak eutrophication, allowing *Daphnia* to allocate high amounts of PUFA into their resting eggs. The high amounts of long‐chain PUFA that we found in resting eggs of all time periods support the idea that these nutrients are required to ensure embryonic development and offspring survival. Among PUFA, especially EPA has been shown to be crucial for growth and reproduction (Becker & Boersma, 2005; von Elert, 2002; Martin‐Creuzburg et al., 2010; Müller‐Navarra et al., 2004). The EPA concentration we found in resting eggs of *Daphnia galeata*/*longispina* was on average 38.2 ng/µg dry weight (ranging from 9.4 to 75.9 ng/µg dry weight). In resting eggs produced on the EPA‐deficient green alga *Scenedesmus obliquus* in the laboratory, EPA concentrations of 2.4 ng/µg (*Daphnia pulicaria*) and ~1.6 ng/µg (*Daphnia magna*) dry weight have been reported (Abrusán et al., 2007; Putman et al., 2015). On a diet rich in EPA (the eustigmatophyte *Nannochloropsis limnetica*), EPA allocation into *D. magna* resting eggs has been shown to be significantly higher (~16.5 ng/µg), but still lower than the maximum values we report here. In *D. magna* resting eggs isolated from the upper 5–10 cm of the sediment of a small Belgian pond, EPA concentrations of ~12.5 ng/µg have been found. Taken together, these data suggest that resting eggs deposited in Lake Constance sediments were produced on diets containing low proportions of green algae and cyanobacteria and high proportions of diatoms, as the latter are typically rich in EPA (Ahlgren et al., 1990; von Elert, 2002; Taipale et al., 2013). This is supported by the long‐term phytoplankton data, showing that the phytoplankton community was always dominated by diatoms, even throughout peak eutrophication (25%–50% of total summer biovolume). The situation might be different, however, in lakes in which the phytoplankton community is dominated by algae providing less long‐chain PUFA, such as green algae or cyanobacteria, potentially resulting in insufficient PUFA allocation into *Daphnia* resting eggs.

*Daphnia* resting eggs deposited for long time periods in the sediment are subject to a process of aging. This aging process becomes evident when resting eggs of different ages are hatched in the laboratory. Older resting eggs typically need longer to hatch and the overall hatching success is reduced as compared to more recently deposited eggs. In Lake Constance, *Daphnia* resting eggs hatch in the order of deposition in the sediment, that is, most recent eggs hatch first, most ancient ones hatch last (personal observation). Resting eggs isolated from Lake Constance sediments that are older than approximately 40 years rarely hatch. The reasons for this ageing process are still unclear. The data we provide here suggest that this is not related to temporal changes in nutrient concentrations because we did not detect a decrease of nutrients with time, that is, age of the resting eggs.

Eutrophication of Lake Constance resulted in changes in the *Daphnia* species composition (Brede et al., 2009; Straile & Geller, 1998). Between 1945 and 2010, two different *Daphnia* species inhabited Upper Lake Constance, *D. longispina* (formerly termed *D. hyalina*) and *D. galeata*. In the first half of the last century, only *D. longispina* occurred in Upper Lake Constance. *Daphnia galeata* invaded Upper Lake Constance in the late 1950s, presumably with the onset of eutrophication (Straile & Geller, 1998). Nowadays, both species and their hybrids are found in the lake, albeit the abundance of *D. galeata* is decreasing in recent years (Straile, 2015). Resting eggs from *D. galeata*, *D. longispina*, and their hybrids could not be distinguished morphologically and our samples thus may have consisted of resting eggs from both species and their hybrids. In general, however, ephippia from *D. longispina* seem to be underrepresented in the sediment of Upper Lake Constance and ephippia deposited after 1960 presumably originate exclusively from *D. galeata* (Brede et al., 2009; Jankowski & Straile, 2003). We thus assume that the majority of resting eggs in our samples originated from *D. galeata*. Only the resting eggs from the two earliest time periods that we analyzed here (45–50, 55–60) most likely originated from *D. longispina*, because this species was the only documented *Daphnia* species in Upper Lake Constance until 1956. We cannot exclude interspecific differences in nutrient allocation into resting eggs. However, the few samples that we were able to analyze from 1945 to 1950 and 1955–1960 do not stick out of our data set or within our GAMs on physiological important PUFAS. Nevertheless, they roughly group together in the PCA, and we thus assume that this potential shift in species might have affected the interpretation of our results. Scores on PC1 differ significantly between resting eggs produced almost exclusively by *D. longispina* (1945–1960) and those produced in other times spans (1965–2010). Recently, presumably in 2014, a third *Daphnia* species, *D. cucullata*, appeared in Upper Lake Constance. However, no resting eggs of *D. cucullata* were found in the egg bank yet. Resting eggs of *D. cucullata* are smaller than the resting eggs of the other species and can be distinguished morphologically from the eggs of other species. Thus, a contribution of *D. cucullata* resting eggs to our samples can be excluded. Our study design is limited by the number of resting eggs that we were able to isolate from time spans older than 1950, resulting in unequal sample sizes. Sediment cores taken from undisturbed deeper areas of Upper Lake Constance are clearly laminated and the potential bias associated with dating and partitioning of the sediment cores is presumably rather low.

## 5 CONCLUSION

Nutrient allocation into *Daphnia* resting eggs seems to be a hardly affected by temporal changes in trophic state and the associated changes in food quantity (i.e., phytoplankton biovolume) and quality (i.e., relative proportions of phytoplankton taxonomic groups). This suggests some sort of homeostatic nutrient regulation within the eggs to ensure offspring survival and that the essential nutrient supply was always sufficient to maintain this homeostasis. In the few cases where we found significant changes in nutrient concentrations over time (C and N), changes could not be linked to eutrophication. Nonetheless, it would be interesting to conduct a similar study in a eutrophic lake with more intense and longer lasting cyanobacterial blooms to assess whether this would affect the allocation of essential lipids (sterols and long‐chain PUFA) into resting eggs. We conclude that the allocation of some nutrients into resting eggs is highly conserved (P, PUFA, and sterols) and mostly resilient toward changes in lake trophic state, while the allocation of other nutrients (C and N) seems to be less tightly regulated and thus affected by environmental changes.

## CONFLICT OF INTEREST

The authors declare that they have no conflict of interest.

## AUTHOR CONTRIBUTION

JIN and DMC designed the study. MW organized the sampling of sediment cores and MW, JIN, CK, and DMC processed them. JIN and CK isolated the eggs and analyzed the samples. JIN and DS analyzed the data. JIN and DMC prepared the manuscript with contributions from all coauthors.

## Data Availability

Nutrient allocation data from resting eggs are available at Dryad: https://doi.org/10.5061/dryad.7d7wm37qw.
