# Supplementary material for: Resilience to changes in lake trophic state: Nutrient allocation into Daphnia resting eggs
Source: Ecol Evol. 2019 Oct 29;9(22):12813–25. doi: 10.1002/ece3.5759 (PMC6875673; doi:10.1002/ece3.5759)
Supplement: Supplementary file 1 [file ECE3-9-12813-s001.pdf]

Supplementary material to:
Resilience to changes in lake trophic state: nutrient allocation into *Daphnia* resting eggs

Jana Isanta Navarro, Carmen Kowarik, Martin Wessels, Dietmar Straile, Dominik Martin-Creuzburg

Figure	dependent variable	independent variable	EDF	Ref.DF	F-value	p-value
2	dryweight	time	1	1	0.04	0.843
3a	C	time	1.25	1.46	3.619	0.0455
3b	N	time	2.159	2.667	4.54	0.0195
3c	P	time	1	1	0.874	0.364
3d	C	tpmix	2.764	3.173	2.349	0.129
3e	N	tpmix	2.428	2.847	4.425	0.0342
4a	C:P	time	1	1	0.001	0.978
4b	C:N	time	2.35	2.875	3.68	0.0321
4c	N:P	time	1	1	0	0.998
4d	C:N	tpmix	1.351	1.609	3.343	0.051
5a	totalFA	time	1	1	0.008	0.931
5b	PUFA	time	1	1	0.059	0.81
6a	ALA	time	1.024	1.048	0.009	0.919
6b	EPA	time	1	1	2.411	0.138
6c	LIN	time	1	1	0.259	0.617
6d	ARA	time	1	1	1.199	0.288
8a	Cholesterol	time	1	1	2.494	0.131
8b	22-dehydrochol	time	1	1	0.493	0.493
8c	total sterol	time	1	1	2.291	0.147
